# Using Catalytic Models to Interpret Age-Stratified Lyme Borreliosis Seroprevalence Data: Can This Approach Help Provide Insight into the Full Extent of Human Infection Occurring at the Population Level?

**DOI:** 10.3390/microorganisms12122638

**Published:** 2024-12-19

**Authors:** Andrew Vyse, Emily Colby

**Affiliations:** 1 Medical Affairs, Pfizer UK Ltd., Tadworth, Surrey KT20 7NS, UK; 2 Vaccines and Antivirals Medical Affairs, Pfizer US Commercial Division, New York, NY 10001-2192, USA; emily.colby@pfizer.com

**Keywords:** Lyme borreliosis, infection, seroprevalence, catalytic models

## Abstract

Diagnosis of Lyme borreliosis (LB) is prone to under ascertainment with the true extent of infection unknown. Cross sectional age-stratified population-based serological survey data may provide insight into this issue. Using data from a previously published Dutch seroprevalence study, we describe the application of catalytic models to make estimates of the annual extent of LB infection. A common assumption when using catalytic models is that IgG is protective and immunity is lifelong. However, human IgG produced in response to natural LB infection does not protect against subsequent infection and its duration may be limited. Individuals were thus assumed to be continually susceptible to LB infection, with a range of scenarios used that varied the length of time that IgG may remain detectable, from 5 years post-infection to lifelong. The possibility that IgG may remain detectable for longer in adults than in children was also explored. Estimates for the annual number of LB infections occurring in the Dutch population ranged from 163,265 (95%CI 130,150–201,723) when assuming IgG remains detectable for only 5 years post-infection to 26,209 (95%CI 17,159–36,557) when assuming IgG is lifelong.

## 1. Introduction

Lyme borreliosis (LB) is a tick-borne disease that occurs following infection with *Borrelia burgdoferi sensu lato* (Bbsl) bacteria and is considered the most common zoonotic disease in the Northern Hemisphere. Human infection follows the bite and subsequent blood-feeding of a Bbsl-infected Ixodes tick. Early symptoms of Bbsl infection are often influenza-like and may be accompanied by an erythema migrans (EM) rash. Such early symptoms can usually be successfully treated with antibiotics. However, if left untreated, systemic disease with severe manifestations affecting the skin, joints, the nervous system and heart can occasionally occur [1,2].

While the incidence of LB occurring in the United States and Europe is substantial, the true extent of human infection with Bbsl at the population level remains unknown, as many LB cases go unrecognized due to inconsistent and incomplete methods of ascertainment or because medical attention is not sought or needed [1,3,4]. Furthermore, initial infection with Bbsl can be asymptomatic [5,6]. With a candidate vaccine to help protect against Bbsl infection now in late development [7], it is becoming important to better understand the full extent to which infection is occurring annually at a country population level and the full extent of the public health need for such a vaccine.

Given that LB case detection is prone to under ascertainment and will not capture asymptomatic infection, population-based seroprevalence studies may provide useful insight to help better understand the full extent of Bbsl infection occurring at a country population level [2,8,9]. Immunoglobulin G (IgG) antibody is generated following antigenic stimulation by a pathogen, and this provides a lasting marker of both prior symptomatic and asymptomatic infection reflecting the totality of infection occurring. LB seroprevalence studies using human sera may therefore help better understand the full extent to which exposure to Bbsl is occurring, giving insight into the size of the population potentially at risk of developing symptoms.

Cross-sectional age-stratified serological surveys measuring antigen-specific IgG show how prevalence of a previous infection changes as age, and therefore cumulative risk of acquiring an infection, increases. Catalytic models can be applied to such age-stratified IgG seroprevalence data to determine the average rate at which susceptible individuals acquire infection, referred to as the force of infection. Catalytic models and the force of infection were first described in the 1930s, and their application to age-stratified IgG seroprevalence data from serosurveys considered to reflect the general population has been used extensively to help estimate the incidence of various common human infections, particularly those occurring in childhood [10,11]. While insight from population based seroprevalence studies is now starting to be used to help estimate the incidence of LB and understand the extent to which it is being under ascertained [8,9], to date catalytic models have not yet been applied to LB seroprevalence data. In this article, we explore the application of catalytic models to some recently published LB serological data from a population-based seroprevalence survey undertaken in the Netherlands [12]; we estimate the annual extent of Bbsl infection that this approach suggests might be occurring in the Dutch population.

## 2. Materials and Methods

Cross-sectional LB seroprevalence data were taken from Supplementary Table S1 in a published serosurvey reflecting the Dutch general population using sera obtained in 2016/17 [12]. Sera were tested using a standard two-tier testing strategy; sera were initially tested with the C6 ELISA (Immunetics, Boston, MA, USA), and positive and equivocal results were confirmed with the *recom*Line Borrelia immunoblot (Mikrogen, Neuried, Germany). Available seroprevalence data were stratified into eighteen 5-year age groups (from ages < 5 years to 85–89 years) with the number of sera tested and proportions testing IgG positive and IgG negative for Bbsl presented in each age group.

Catalytic models were used to model these LB seroprevalence data as previously described [13]. A generalization of the relationship for an age-independent force of infection was employed, where the average annual age-specific force of infection [λ_a_] is related to the seroprevalence at age *a*, *P_[a]_*, using the equation below:

$$P_{\left[a\right]}=1-\exp-[\int_{0}^{a}\lambda \left(a\right)da]$$

**Figure 1:**
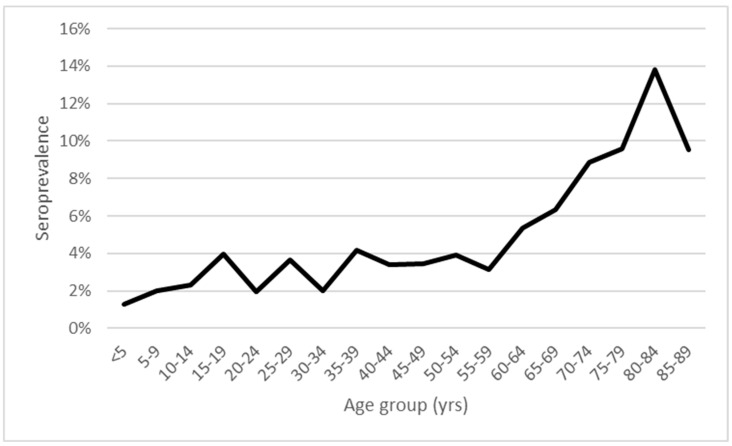
Observed seroprevalence by age group constructed using data presented by Hoeve-Bakker et al. 2023 Supplementary Table S1 [12].

Estimates for λ_a_ were made in three age categories: <15 years, 15–59 years and ≥60 years using a maximum likelihood technique. The choice of these age categories was motivated by the shape of the observed age-stratified seroprevalence curve and reflects those age points where a substantial overarching change in the gradient occurred (Figure 1). The analysis was undertaken in Microsoft Excel 365 using the solver function to estimate an optimal value for λ_a_ in each of the three age categories. The maximum likelihood technique for fitting the model to the observed data utilized the number and proportion of samples presented in the published serosurvey that tested seropositive and seronegative in each of the eighteen 5-year age groups to minimize the deviance between the modeled and observed seroprevalence [13,14]. Likelihood-based 95% confidence intervals for λ_a_ in each age category were obtained by finding the maximum and minimum values for which the deviance, minimized with respect to the other parameters, was within 3.84 of the minimum [13]. Insight into how well the model fitted the observed data was obtained by comparing the model deviance to the degrees of freedom (the number of data points minus the number of model parameters), with a deviance ≤ the number of degrees of freedom considered to reflect a good fit [13]. The model fit to the observed data is also presented graphically and can be assessed visually. Publicly available age-stratified Dutch national population data in 2017 [15] were used in conjunction with estimates for λ_a_ in each age category to calculate the number of infections occurring annually in the Dutch population.

A commonly used assumption when applying catalytic models to seroprevalence data is that IgG is long lasting and confers lifelong immunity [11]. However, human IgG produced in response to natural infection with Bbsl is not considered protective with mechanisms available to the Bbsl bacterium to help it avoid the immune response [16]. Reinfection is therefore possible following a subsequent bite and blood feeding event by a Bbsl-infected tick. Individuals were thus assumed to be continually susceptible to Bbsl infection across the full life course. Furthermore, there is some evidence that IgG generated following human infection with Bbsl may only be detectable in serological assays for a more limited period rather than being very long lasting or lifelong [9,17,18,19,20,21,22,23]. While this period is likely to still reflect a considerable number of years, its average (e.g., median) duration is not well understood. The small number of relevant studies that currently provide some insight into duration suggest a wide range of estimates from <10 years to >30 years. The length of time that LB IgG may remain detectable for, with respect to the time of sample collection to the time when initial infection occurred for each age group, was therefore varied in model scenarios using assumptions that this may be lifelong, 30 years, 20 years, 10 years or 5 years. There is also some evidence that Bbsl IgG is possibly detectable for longer in adults than in children [17,18]. Additional scenarios were therefore explored, assuming this to be 10 years, 15 years and 20 years in those aged < 15 years and 20 years and 30 years in those aged ≥ 15 years.

## 3. Results

A total of 5592 sera were tested for Bbsl IgG in the published serosurvey used for this analysis. The number of sera tested in each 5-year age group was substantial for those aged between 5 years and 79 years (ranging from 198 sera in those aged 75–79 years to 460 sera in those aged 20–24 years). However, for those aged < 5 years, 80–84 years and particularly those aged 85–89 years, considerably fewer sera were tested (78, 94 and 21, respectively). The overall observed seroprevalence for all age groups combined was 4.2% with observed seroprevalence increasing with increasing age from 1.3% in those aged <5 years to 13.8% in those aged 80–84 years. Observed seroprevalence initially increased to 4% in those aged 15–19 years but then showed little evidence of any increase until aged 60 years, when observed seroprevalence subsequently then increased steeply with age in those aged 60–84 years to a peak of 13.8% in those aged 80–84 years before then declining to 9.5% in those aged 85–89 years. No seroprevalence data were available for those aged ≥ 90 years. While little overall increase in observed seroprevalence occurred in ages 15–59 years, the observed seroprevalence in those aged 15–39 years was not stable and fluctuated from 2% to 4% (Figure 1).

**Figure 2:**
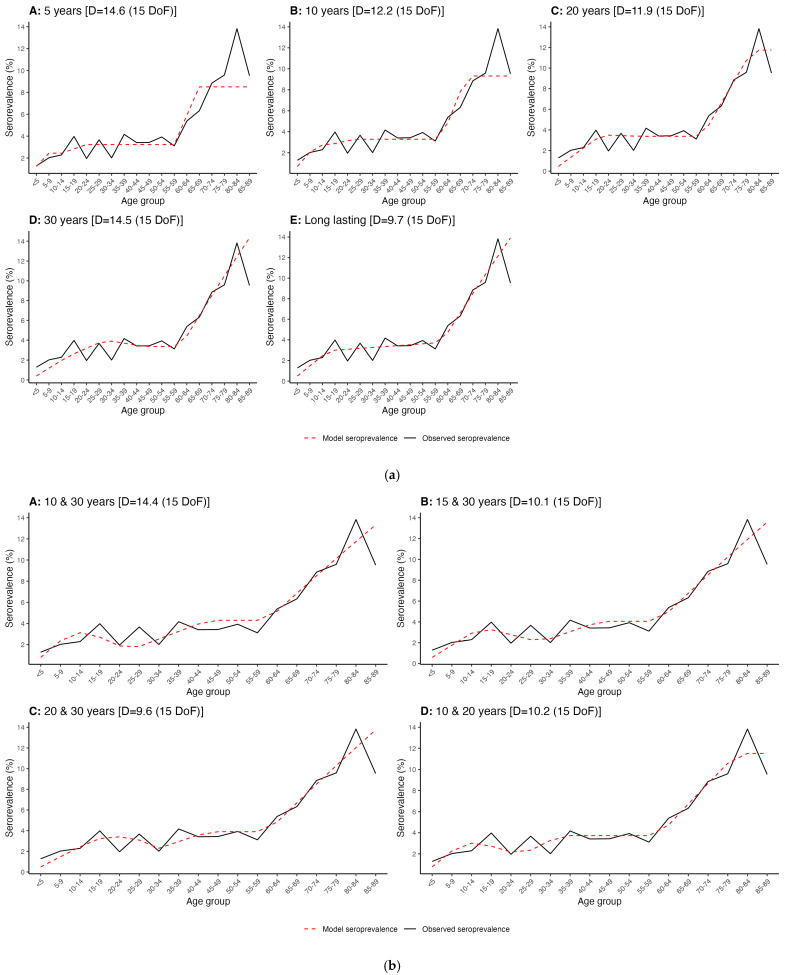
(**a**). Modeled seroprevalence compared to observed seroprevalence (model fit to data) assuming duration of IgG post-infection with respect to time of sample collection is (**A**) 5 years; (**B**) 10 years; (**C**) 20 years; (**D**) 30 years; (**E**) lifelong. (**b**). Modeled seroprevalence compared to observed seroprevalence (model fit to data) assuming duration of IgG post-infection with respect to time of sample collection varies by age and is (**A**) 10 years in those aged <15 y and 30 years in those aged ≥15 y; (**B**) 15 years in those aged <15 y and 30 years in those aged ≥15 y; (**C**) 20 years in those aged <15 y and 30 years in those aged ≥15 y; (**D**) 10 years in those aged <15 y and 20 years in those aged ≥15 y.

Overall, all the model scenarios used to estimate λ_a_ fitted the serological data well, ranging from a deviance (D) of 9.6 on 15 degrees of freedom (DoF) to a D of 14.6 on 15 DoF depending on the assumptions used for the length of time Bbsl IgG may remain detectable for post-infection (Figure 2a,b). However, the observed seroprevalence fluctuated with age for those aged 15–39 years. While this unstable trend was not precisely captured by the model, it did nevertheless describe a good approximation for the overall observed seroprevalence trend in this age range. The model scenarios that assumed duration of Bbsl IgG was longer in adults compared to children also better reflected the observed fluctuation in observed seroprevalence for this age group. The model also did not specifically capture the substantial decline in observed seroprevalence that occurred for those aged 85–89 years compared to those aged 80–84 years and generally tended to reflect an average of the observed seroprevalence for those aged 80–89 years. When assuming the duration of Bbsl IgG was 5 years and 10 years, the ability of the scenarios to capture observed seroprevalence in the oldest age groups was also more limited compared to the other scenarios. These scenarios suggested maximum seroprevalence would occur at approximately 70 years of age and then plateau.

Table 1a,b show the force of infection estimates for each age category using the different assumptions for the length of time Bbsl IgG may remain detectable for post-infection. Table 2a,b show the resulting total annual infections at the population level suggested using these assumptions in the different scenarios explored. This ranged from 26,209 (95% CI 17,159–36,557) infections assuming Bbsl IgG is lifelong to 163,256 (95% CI 130,150–201,723) infections when assuming Bbsl IgG is only detectable for 5 years post-infection in all age groups.

**Figure 3:**
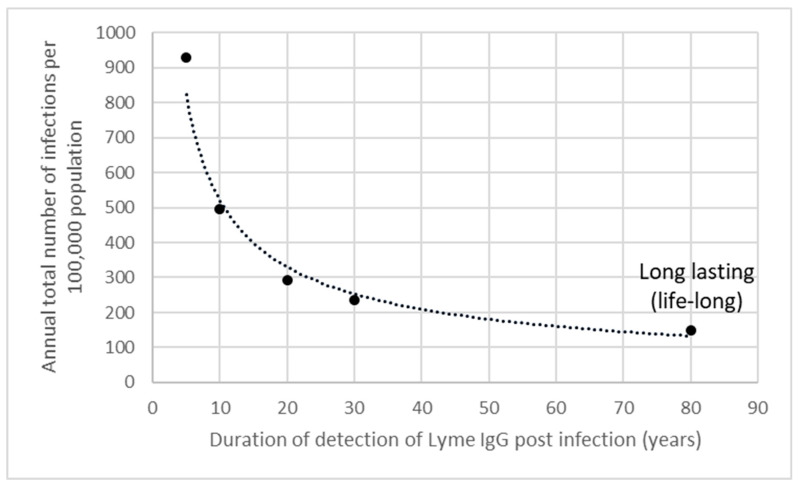
Model estimates for the annual total LB incidence (all age groups combined) by length of time that Lyme borreliosis IgG may remain detectable for post-infection.

Figure 3 shows how the estimated total annual infections of Bbsl infection for all age groups combined increases as the assumption used for the length of time that LB IgG may remain detectable for post-infection decreases, assuming this does not vary by age group. The average life expectancy in the Netherlands (~81 years) was used when Bbsl IgG was assumed to be detectable lifelong post-infection [24]. The scenarios where the time that LB IgG remains detectable for post-infection is longer in adults compared to children gave less variation in the total annual infections estimated. These ranged from 43,372 (95% CI 32,989–55,383) when assuming Bbsl IgG is detectable for 20 years and 30 years post-infection in those aged < 15 years and ≥15 years, to 56,183 (95%CI 43,428–68,699) when assuming Bbsl IgG is detectable for 10 years and 20 years post-infection in those aged < 15 years and ≥15 years.

## 4. Discussion

The observed age-stratified seroprevalence suggested the rate of acquisition of Bbsl IgG initially rises with increasing age in children aged < 15 years with a second more rapid increase with age then occurring in older adults beginning at age 60 years. This is consistent with a recent study that investigated incidence of LB recorded in Dutch primary care across a similar period [2015–2019], which found this was highest in those aged 5–14 years and 60–69 years, respectively; it is also consistent with the bimodal distribution of LB cases that is commonly observed with respect to age [16,25]. This bimodal distribution reflects those age groups with the highest likelihood of exposure to ticks and is consistent with the choice of age groups used for estimating the force of infection in this analysis. However, the overall flatness of the observed seroprevalence curve for the ages 15–59 years suggests very little (if any) infection and subsequent acquisition of Bbsl IgG is occurring in this age group. This observation is reflected by the very low force of infection estimate in those aged 15–59 years when Bbsl IgG is assumed to be very long-lasting (lifelong) with a lower bound 95% confidence interval that included zero. While this age group is generally considered to be at a lower risk of Bbsl infection compared to children and older adults, the incidence of LB cases in this age group recently estimated using Dutch primary care data suggests the force of infection they experience may not be so negligible [25]. One possible explanation for this flatness of the seroprevalence curve is that Bbsl IgG may not be lifelong and may only remain detectable for a limited period following infection [17,18]. The model scenarios investigating periods of shorter Bbsl IgG duration resulted in substantial increases in the force of infection being estimated for those aged 15–59 years but had more limited impact on the force of infection estimated in those aged < 15 years and ≥60 years.

All the model scenarios fitted the observed serological data well, making it difficult to select an obvious optimal candidate scenario. The best fitting scenario (i.e., that giving the lowest deviance) assumed Bbsl IgG remained detectable for 20 years and 30 years post-infection in those aged < 15 years and ≥15 years, respectively, with a marginally better fit than the scenario assuming Bbsl IgG was lifelong. The scenario assuming that Bbsl IgG is lifelong estimated the smallest total annual incidence of infection affecting the Dutch population. This forms a useful baseline reference point reflecting the most conservative approach with this methodology and is approximately 25% higher than the annual incidence of LB cases estimated using Dutch primary care data obtained at a similar time [25]. These seroprevalence data are therefore suggesting there must be at least ~26,000 Bbsl occurring infections annually in the Dutch population.

In contrast, the annual incidence of infection estimated by the scenarios assuming Bbsl IgG is detectable for shorter periods post-infection increased substantially, with the force of infection estimated in those aged 15–59 years being especially sensitive to this. This was particularly the case when the duration of detection was reduced to 5 years and 10 years, where the resulting total annual infections estimated were approximately four times and eight times higher, respectively, than the number of LB cases estimated from Dutch primary care data [25]. A substantial proportion of these infections occurred in those aged 15–59 years, due to the considerably higher force of infection estimates being made by the model scenarios for this age group when using assumptions that Bbsl IgG remains detectable for much more limited periods post-infection. However, these scenarios gave force of infection estimates in those aged 15–59 years that were greater than those estimated in those aged < 15 years. This may be unrealistic given the bimodal nature of observed LB incidence, with peaks typically seen in children and especially older adults. All other model scenarios estimated a force of infection in those aged 15–59 years that was lower than that estimated in those aged < 15 years and therefore more consistent with the age distribution of LB cases observed. Consideration must also be given to the plausibility of the annual estimate of up to 163,256 infections, derived from the model assuming Bbsl IgG remains detectable for only 5 years post-infection. Each year ~1.5 million tick bites are thought to occur in the Netherlands with ~15% of ticks estimated to be carrying Bbsl and a 2–3% probability of becoming infected following a bite [25]. This potentially suggests that an annual total of 45,000 to 67,500 Bbsl infections might be expected to occur in the Dutch population. This is more consistent with estimates from the model scenarios that assumed Bbsl IgG remained detectable for 20 years post-infection and the four model scenarios that assumed the average time that LB IgG remains detectable for is longer in adults compared to children.

There are various limitations and points to consider with this analysis. Firstly, while the serum samples used to generate the seroprevalence data used in the analysis are considered to reflect the general Dutch population, they may still contain some selection bias [12]. Secondly, the time that Bbsl remains detectable for post-infection in serological assays is not well understood at present. This has a significant influence on the incidence of infection subsequently being estimated from these serosurvey data. While there is some evidence that duration is not necessarily lifelong and may be more limited, this aspect is not currently well established. Furthermore, the potential for reinfection suggests Bbsl IgG levels may be periodically boosted with increasing age and thresholds in older age groups may take longer to fall to levels below assay thresholds of detection. This would support Bbsl IgG being detectable for a more limited period post-infection in younger age groups, for which there is some evidence [17,18]. Further research is therefore needed into the length of time Bbsl IgG remains detectable for post-infection with catalytic models possibly needing to be adapted to capture this aspect more accurately. While it is recognized that some Bbsl infection will be latent due to it either being asymptomatic or not considered severe enough for medical attention to be sought or needed, the extent to which this occurs is also currently not well defined [9]. The proportion of Bbsl infections that are likely to be symptomatic therefore requires further research and will help contextualize estimates of total infection made using serosurvey data that are of public health relevance. Furthermore, that reinfection with Bbsl is possible suggests that there may always be some under ascertainment with force of infection estimates made from serological data. It is not possible to identify multiple infections from the serological data used. Seropositive samples therefore were assumed to reflect a single infection event. This issue is likely to be particularly relevant in older age groups where the cumulative risk of tick exposure and likelihood of experiencing more than one Bbsl infection event is greatest.

A common assumption used when applying catalytic models to serological data is that a perfect test was used to generate the data [11]. LB serology often employs a two-tier testing method involving an initial ELISA screen followed by a confirmatory Western blot, as was the case for the serological data used in this analysis. This approach is primarily intended for clinical diagnosis in the individual rather than epidemiological screening for any evidence of past infection in population serosurveys. In the clinical context, a fixed assay cutoff used to determine positivity will likely be biased towards individual diagnosis with some sensitivity sacrificed for specificity so there is a high positive predictive value associated with samples classified as positive. This may result in seroprevalence being under ascertained when such cutoffs are applied to data from population serosurveys, particularly for those samples containing lower levels of Bbsl IgG, which nevertheless may still reflect a prior infection [13,26]. Future LB serosurveys may therefore need to be undertaken using assay cutoffs specifically designed for seroepidemiology [26].

The risk of human exposure to ticks carrying Bbsl may not be in endemic equilibrium. The size and density of tick populations and the proportion carrying Bbsl is known to vary with time and geographical location and is also expanding with climatic and environmental change [27]. Such changing risk of Bbsl infection by time and location has implications for cross-sectional LB seroprevalence studies using samples collected at a single point in time, suggesting the need for more regular serosurveys. Analyses may also need to consider the possibility of age cohort effects, particularly if Bbsl IgG is determined to be very long lasting. Furthermore, this analysis is exclusive to the Netherlands and reflects the local risk of exposure to ticks and subsequent Bbsl infection being experienced by the Dutch population. The risk of human exposure to Bbsl-infected ticks varies geographically between countries and this will be reflected by country-specific age-stratified seroprevalence data [28]. This supports the need for more contemporary individual country-level age-stratified serosurvey data that reflect the general population across the full age range to better understand the extent of infection occurring in different countries. At present, such country-specific seroprevalence data are relatively limited and are becoming historical [28].

Lastly, it is worth considering in more detail the observed seroprevalence in the most elderly aged (aged ≥ 80 years) and in those aged 15–39 years. The observed seroprevalence in those aged 85–89 years was considerably lower than that in those aged 80–84 years. Only a very limited number of sera were tested from those aged 85–89 years, resulting in a lack of precision for the observed seroprevalence estimated in this age group. No seroprevalence data were available for those aged ≥ 90 years. While the observed seroprevalence increased strongly with age from 60–85 years before then declining in those aged 85–89 years, the extent to which the trend in seroprevalence may subsequently change in those aged ≥ 85 years is therefore uncertain (i.e., whether it continues to increase, plateaus or declines) since the observed seroprevalence in this age group is based on a very small sample size. It seems reasonable to assume that the risk of Bbsl infection potentially declines (possibly substantially) in the very elderly as frailty increases and the likelihood of outdoor activity and risk of tick exposure reduces. Future catalytic models may therefore need to include an additional parameter to accommodate a possible lower force of infection in the most elderly. If possible, future serosurveys should try to collect more samples from the very elderly to obtain more robust observed estimates of seroprevalence in these oldest age groups to help better inform this aspect. However, obtaining sufficient sera in this age group is likely to be logistically difficult. Furthermore, the scenarios assuming Bbsl IgG was only detectable for up to 5 years and 10 years post-infection both suggested that a maximum seroprevalence would be achieved by approximately age 70 years in the Dutch population and would then plateau, which correlated poorly with the observed data for those aged 70–84 years. This suggests these scenarios may be unlikely and that Bbsl IgG generally remains detectable for >10 years post-infection. It is also notable that there are considerable sharp fluctuations in observed seroprevalence estimated for those aged 15–39 years despite the large number of sera tested in this age group. This is not apparent for other age groups. A possible explanation is that this may reflect a limitation of the fixed assay cutoffs used and that samples containing lower levels of Bbsl IgG reflect more distant infection that occurred during childhood. Such levels may not always reach the threshold needed to be classified as seropositive [13,26]. This is also an age category where LB incidence is generally observed to be lower compared to other age groups, suggesting existing Bbsl IgG levels are less likely to be boosted by reinfection [16,25]. While this could be an artefact of the serological assay used, there could also be a biological explanation for this phenomenon. More research into the incidence of LB infection and the dynamics of the Bbsl IgG response in this age group specifically may help us better understand the fluctuating nature of the observed seroprevalence in these younger adults. However, it is notable that those model scenarios that assumed duration of Bbsl IgG was longer in adults compared to children did better reflect the fluctuation in observed seroprevalence for those aged 15–39 years. This suggests that differing Bbsl IgG duration by age group could be an important factor contributing to this observed fluctuation in seroprevalence in younger adults.

## 5. Conclusions

Interpreting age-stratified LB seroprevalence data from serosurveys that reflect the general population with catalytic models has potential to provide insight into the extent of annual infection with Bbsl that is occurring. This can complement other approaches that utilize other data sources to estimate incidence of LB cases [29]. Catalytic models may need adapting as the understanding of Bbsl IgG increases, particularly how long it may remain detectable for post-infection using current serological assays. Regular serosurveys may also need to be undertaken to account for the fluctuating dynamics of tick populations, climate change and the resulting risk of human infection with Bbsl. However, this analysis suggests the annual incidence of LB infection affecting the Dutch general population is substantial and may be considerably larger than that indicated by existing data from primary care.

## Figures and Tables

**Table 1:** (a). Force of infection estimates (95%CI) with increasing length of time that Lyme IgG may remain detectable for post-infection. (b). Estimates of the total annual infections (95%CI) with increasing time periods that Lyme borreliosis IgG may remain detectable for post-infection.

**a**					
**Age Group (y)**		**Duration of Detection for Lyme Borreliosis IgG**			
	**5 Years**	**10 Years**	**20 Years**	**30 Years**	**Lifelong**
**<15**	0.005 (0.003–0.008)	0.0028 (0.0018–0.0041)	0.0019 (0.0013–0.0026)	0.0016 (0.0012–0.0022)	0.0020 (0.0017–0.0024)
**15–59**	0.007 (0.005–0.008)	0.0034 (0.0028–0.0041)	0.0018 (0.0014–0.0021)	0.0012 (0.0010–0.0015)	0.0002 (0–0.00039)
**60+**	0.018 (0.014–0.022)	0.0098 (0.0070–0.0120)	0.0063 (0.0049–0.0079)	0.0056 (0.0042–0.0071)	0.0041 (0.0028–0.0057)
**b**					
		**Duration of Detection for Lyme Borreliosis IgG**			
	**5 Years**	**10 Years**	**20 Years**	**30 Years**	**Lifelong**
**Annual** **infections**					
**Age < 15 y**	13,422 (8002–20,641)	7525 (4767–11,066)	4918 (3365–6829)	4302 (3090–5737)	5410 (4496–6419)
**Age 15–59 y**	66,700 (55,049–80,433)	34,093 (27,967–41,017)	17,538 (14,202–21,300)	11,642 (9192–14,419)	1871 (0–3987)
**Age > 60 y**	82,834 (67,099–100,649)	45,543 (36,494–55,901)	29,096 (22,601–36,581)	25,734 (19,430–33,000)	18,928 (12,663–26,154)
**Total annual infections**	163,256 (130,150–201,723)	87,161 (69,228–107,984)	51,552 (40,168–64,710)	41,678 (31,712–53,156)	26,209 (17,159–36,557)
**Total per 100,000 population**	929 (741–1148)	496 (394–615)	293 (229–368)	237 (181–303)	149 (98–208)

**Table 2:** (a). Force of infection estimates (95%CI) with time periods that Lyme borreliosis IgG may remain detectable for post-infection exploring scenarios where this is longer in adults compared to children. (b). Estimates of the total annual infections (95%CI) exploring scenarios where the time period that Lyme borreliosis IgG may remain detectable for post-infection is longer in adults compared to children.

**a**				
**Age Group (y)**	**Duration of Detection for Lyme Borreliosis IgG**			
	**10 y (Aged < 15 y) 30 y (Aged ≥15 y)**	**15 y (Aged < 15 y) 30 y (Aged ≥15 y)**	**20 y (Aged < 15 y) 30 y (Aged ≥ 15 y)**	**10 y (Aged < 15 y) 20 y (Aged ≥ 15 y)**
**<15**	0.0032 (0.0022–0.0046)	0.0024 (0.0017–0.0034)	0.0020 (0.0014–0.0028)	0.0031 (0.0020–0.0045)
**15–59**	0.0015 (0.0013–0.0018)	0.0014 (0.0012–0.0017)	0.0014 (0.0011–0.0017)	0.0019 (0.0016–0.0023)
**60+**	0.0051 (0.0038–0.0067)	0.0052 (0.0039–0.0068)	0.0053 (0.0040–0.0069)	0.0062 (0.0048–0.0072)
**b**				
**Disease burden**	**Duration of detection for Lyme borreliosis IgG**			
	**10 y (Aged < 15 y) 30 y (aged ≥15 y)**	**15 y (Aged < 15 y) 30 y (aged ≥15 y)**	**20 y (Aged < 15 y) 30 y (aged ≥15 y)**	**10 y (Aged < 15 y) 20 y (aged ≥15 y)**
**Annual infections (n)**				
**Age < 15 y**	8621 (5706–12,303)	6425 (4369–8966)	5357 (3720–7344)	8297 (5427–11,939)
**Age 15–59 y**	14,940 (12,365–17,813)	14,089 (11,554–16,927)	13,515 (11,014–16,326)	19,368 (15,958–23,207)
**Age > 60 y**	23,612 (17,413–30,786)	24,140 (17,910–31,335)	24,500 (18,255–31,713)	28,518 (22,043–33,553)
**Total annual infections (n)**	47,173 (35,484–60,902)	44,654 (33,833–57,228)	43,372 (32,989–55,383)	56,183 (43,428–68,699)
**Total annual infections per 100,000 population**	296 (202–347)	255 (193–326)	247 (188–316)	320 (248–392)

## Data Availability

All data used in this study are freely available in the public domain.
